# Metagenomic data from surface seawater of the east coast of South Korea

**DOI:** 10.1038/s41597-023-02556-7

**Published:** 2023-09-22

**Authors:** Yeonjung Lim, Seung-Jo Yang, Ilnam Kang, Jang-Cheon Cho

**Affiliations:** 1 Center for Molecular and Cell Biology, Inha University, Inha-ro 100, Incheon, 22212 Republic of Korea; 2 CJ Bioscience, Inc., Sejong-daero 14, Seoul, 04527 Republic of Korea; 3 Department of Biological Sciences and Bioengineering, Inha University, Inha-ro 100, Incheon, 22212 Republic of Korea

**Keywords:** Metagenomics, Microbial ecology

## Abstract

The East Sea, also known as the Sea of Japan, is a marginal sea located in the western Pacific Ocean, displaying comparable characteristics to Earth’s oceans, thereby meriting its recognition as a “miniature ocean”. The East Sea exhibits a range of annually-recurring biogeochemical features in accordance with seasonal fluctuations, such as phytoplankton blooms during the spring and autumn seasons. Despite ongoing monitoring efforts focused on water quality and physicochemical parameters, the investigation of prokaryotic assemblages in the East Sea, encompassing seasonal variations, has been infrequently pursued. Here, we present a monthly time-series metagenomic dataset spanning a one-year period in 2009, obtained from surface (10 m) seawater samples collected off the coast of the East Sea. The dataset encompasses 12 metagenomes, amounting 195 Gbp, with 14.73–22.52 Gbp per sample. This dataset is accompanied by concurrently measured physicochemical parameters. Our anticipation is that these metagenomes will facilitate extensive investigations aimed at elucidating various aspects of the marine microbial ecosystems in the East Sea.

## Background & Summary

Metagenomics has emerged as a fundamental approach in marine environmental studies, deciphering the intricacies and diversity of microbial communities in the oceans and their environmental interplay^1–4^. This technology has facilitated the discovery of hitherto unknown microbes^5–7^, genes^8–10^, and metabolic pathways^11,12^, thereby considerably enriching our understanding of marine biodiversity and ecosystem function. Additionally, metagenomic techniques present opportunities for new biotechnological discoveries, such as enzyme development for industry and the identification of bioactive compound sources^13–15^. The continuous evolution of metagenomic tools can substantially augment our comprehension of Earth’s ecosystems, necessitating the generation and efficient exploitation of purpose-aligned metagenomic data.

The East Sea, which is also referred to as the Sea of Japan, is a semi-enclosed marginal sea situated in the western Pacific Ocean, and is colloquially termed a “miniature ocean” owing to its resemblance to the global oceans^16^. One of the prominent features of the East Sea is the Tsushima Warm Current (TWC), which originates from the south and is a major driver in shaping the region’s oceanic circulation by modulating water temperature, salinity, and nutrient dispersion. The substantial nutrient influx via these currents, in tandem with coastal upwelling^17,18^, results in high primary productivity, especially associated with the annual cycle of spring (April-June) and autumn (October-November) phytoplankton blooms^19–21^. Such recurrent biogeochemical fluctuations necessitate regular assessments to understand their impact on the microbial ecology of the East Sea, as shown by studies in the North Sea^22^.

Numerous long-term studies from the East Sea have been undertaken via programs including the Circulation Research in the East Asian Marginal Sea (CREAMS), CTD station operations^23,24^ or satellite colour measurements^20,25^. To date, however, there appears to be a lacuna in studies specifically focusing on the monthly variation of microbial community structure in the East Sea, which are indicative of seasonal changes in environmental parameters such as chlorophyll a concentration. Previous microbial investigations in the East Sea have primarily concentrated on the deep-sea sediments or methane hydrate-containing sediments^26–29^. In addition, a metagenomic study has been undertaken to assess the influence of environmental determinants on the spatial distribution of pelagic bacteria in the East Sea, albeit limited to a bimonthly scale during the summer and winter months^30^.

**Fig. 1:**
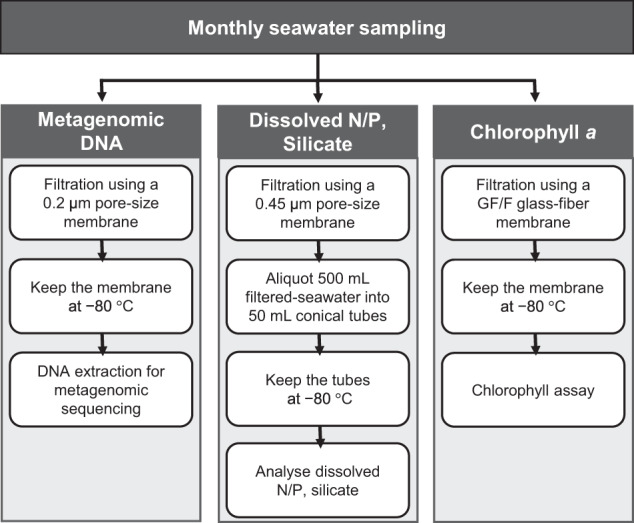
Schematic diagram of the processing of seawater samples.

In this study, we present a one-year (January to December 2009) monthly metagenomic dataset derived from the East Sea’s coastal waters. Seawater samples were filtered using a 0.2 µm pore-size membrane and subsequently cryopreserved at −80 °C until DNA extraction, followed by sequencing via the Illumina HiSeq platform. The physicochemical characteristics of the water samples were concurrently measured to infer the environmental factors influencing the microbial community. The schematic diagram illustrating the methodology used for generating this dataset is presented in Fig. 1.

Our selected sampling locations are of considerable scientific import due to the successful *in situ* isolation of a plethora of bacterial strains belonging to major marine bacterial clades, including SAR11, oligotrophic marine gammaproteobacteria (e.g., SAR92, OM60), OM43, and SAR116^31–33^. Therefore, the creation of this monthly metagenomic repository can offer an asset for investigating prokaryotic assemblages of temperate coastal seas through both culture-dependent and culture-independent methodologies. Furthermore, metagenomic analyses may unveil previously uncultured microbial species and suggest potential cultivation strategies. This exhaustive insight into the microbial community of this “miniature ocean” holds promise for fostering a deeper understanding of global marine ecosystems.

## Methods

### Sampling process

**Fig. 2:**
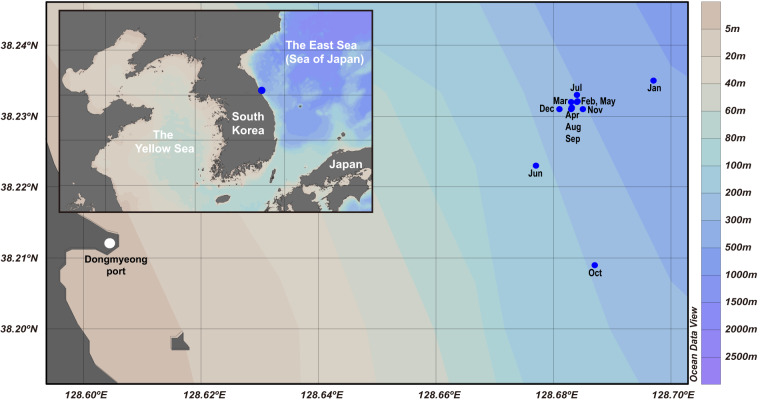
Map of the sampling stations. Sampling stations of each month are indicated as blue dots with three-letter abbreviations of months. Inset at the upper left shows the approximate location of the sampling stations in the East Sea.

Seawater samples were collected monthly off the coast of the East Sea, in proximity to Sokcho, Korea, throughout the year 2009. Sampling was executed approximately 8 km from Dongmyeong Port (Fig. 2 and Table 1), with the exact location of sampling stations subject to minor variations due to atmospheric conditions. Approximately 10 litres of surface seawater samples were collected from a depth of 10 m using a Niskin sampler (General Oceanics, Inc., USA) and were transported to the laboratory in an ice-cooled box. The water samples (6 litres; 6 replicates of each 1 litre) were filtered through 0.2 μm pore-size polyethersulfone membrane filter (47 mm in diameter, Supor®, Pall, USA) for DNA extraction. Additionally, 1 litre was filtered using a 47 mm GF/F glass-fiber filter (Whatman, USA) to analyse chlorophyll *a*. All filters were stored at −80 °C until further processing. The residual volume was filtered employing a 0.45 μm pore-size cellulose ester membrane filter (Advantec, Japan), aliquoted into 50 ml conical tubes (Falcon, USA), and preserved at −80 °C, to be later used for the analysis of environmental parameters including dissolved inorganic ions (ammonium, nitrite + nitrate, phosphate, and silicate). Temperature and salinity of the water samples were measured onboard using a YSI 30 (YSI Inc., USA). The total cell count was conducted using epifluorescence microscopy (Nikon 80i, Nikon, Japan), enumerating DAPI-stained cells (Table 2).

**Table 1:** Data availability of the metagenomic sequences from the East Sea, South Korea.

BioProject	SRA Study	BioSample	SRA Sample	Collection date	GPS	SRA Experiment	Library Name	SRA Run	# of Spots	Total Bases (Gb)
PRJNA877782	SRP396155	SAMN30722872	SRS15031509	2009-01-16	38.235 N 128.697 E	SRX17478415	ES_Meta_2009_01	SRR21474786	32,470,556	16.30
PRJNA877782	SRP396155	SAMN30722873	SRS15031510	2009-02-18	38.232 N 128.684 E	SRX17478416	ES_Meta_2009_02	SRR21474785	29,755,955	14.94
PRJNA877782	SRP396155	SAMN30722874	SRS15031513	2009-03-31	38.232 N 128.683 E	SRX17478419	ES_Meta_2009_03	SRR21474782	29,430,338	14.77
PRJNA877782	SRP396155	SAMN30722875	SRS15031514	2009-04-30	38.231 N 128.683 E	SRX17478420	ES_Meta_2009_04	SRR21474781	44,857,432	22.52
PRJNA877782	SRP396155	SAMN30722876	SRS15031515	2009-06-05	38.232 N 128.684 E	SRX17478421	ES_Meta_2009_05	SRR21474780	29,347,473	14.73
PRJNA877782	SRP396155	SAMN30722877	SRS15031516	2009-06-25	38.223 N 128.677 E	SRX17478422	ES_Meta_2009_06	SRR21474779	29,356,194	14.74
PRJNA877782	SRP396155	SAMN30722878	SRS15031517	2009-07-31	38.233 N 128.684 E	SRX17478423	ES_Meta_2009_07	SRR21474778	29,887,630	15.00
PRJNA877782	SRP396155	SAMN30722879	SRS15031518	2009-08-21	38.231 N 128.683 E	SRX17478424	ES_Meta_2009_08	SRR21474777	29,363,801	14.74
PRJNA877782	SRP396155	SAMN30722880	SRS15031519	2009-09-30	38.231 N 128.683 E	SRX17478425	ES_Meta_2009_09	SRR21474776	34,963,503	17.55
PRJNA877782	SRP396155	SAMN30722881	SRS15031520	2009-10-30	38.209 N 128.687 E	SRX17478426	ES_Meta_2009_10	SRR21474775	29,873,136	15.00
PRJNA877782	SRP396155	SAMN30722882	SRS15031511	2009-11-30	38.231 N 128.685 E	SRX17478417	ES_Meta_2009_11	SRR21474784	34,465,700	17.30
PRJNA877782	SRP396155	SAMN30722883	SRS15031512	2009-12-29	38.231 N 128.681 E	SRX17478418	ES_Meta_2009_12	SRR21474783	34,627,707	17.38

**Table 2:** Physicochemical parameters of seawater samples collected monthly.

Month	Date	Depth (m)	Total cell (cells mL^−1^)	Temperature (°C)	salinity (‰)	Chlorophyll *a* (μg L^−1^)	PO_4_^3−^ (μmol L^−1^)	NO_3_^−^ +NO_2_^−^ (μmol L^−1^)	SiO_2_ (μmol L^−1^)	NH_4_^+^ (μmolL^−1^)
**Jan**	2009-01-16	10	5.61 × 10^5^	10.9	33.6	0.13	0.014	0.055	0.416	n/d
**Feb**	2009-02-18	10	4.55 × 10^5^	9.9	34.3	0.16	0.023	0.102	0.771	0.004
**Mar**	2009-03-31	10	9.10 × 10^5^	12.2	34.3	0.07	0.021	0.082	0.660	0.005
**Apr**	2009-04-30	10	1.10 × 10^6^	11.5	34.3	0.55	0.016	0.030	0.418	0.003
**May**	2009-06-05	10	1.52 × 10^6^	11.1	34.0	1.00	0.015	0.004	0.444	0.001
**Jun**	2009-06-25	10	1.10 × 10^6^	16.6	33.6	0.21	0.010	n/d*	0.159	0.001
**Jul**	2009-07-31	10	8.66 × 10^5^	14.3	33.2	0.08	0.009	n/d	0.115	n/d
**Aug**	2009-08-21	10	6.12 × 10^5^	15.8	33.4	0.07	0.009	n/d	0.181	0.004
**Sep**	2009-09-30	10	8.70 × 10^5^	20.6	32.9	0.05	0.010	n/d	0.132	n/d
**Oct**	2009-10-30	10	9.71 × 10^5^	18.1	33.2	0.40	0.017	0.068	0.749	n/d
**Nov**	2009-11-30	10	8.35 × 10^5^	11.6	33.6	0.28	0.011	0.027	0.291	0.010
**Dec**	2009-12-29	10	5.39 × 10^5^	12.0	34.0	0.19	0.021	0.085	0.628	0.004

*Numeric values less than or equal to zero are denoted as ‘n/d’

### Biogeochemical analyses

**Fig. 3:**
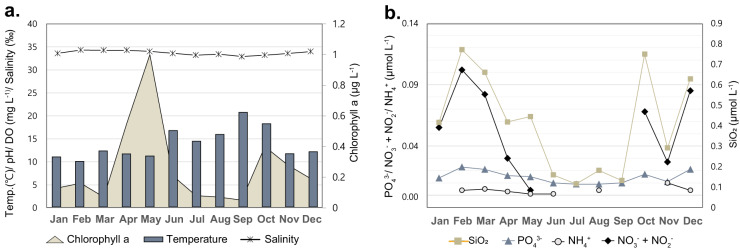
Physicochemical parameters of seawater samples collected monthly.

Chlorophyll *a* was extracted from GF/F glass-fiber filters using 90% aqueous acetone (v/v) at 4 °C overnight. The extraction solution was centrifuged for 10 min at 2,000 rpm, and the supernatants were analysed via a fluorometer (10 AU, Turner Designs, USA). Concentrations of inorganic nutrients, including NO_2_^−^, NO_3_^−^, NH_4_^+^, PO_4_^3−^, and SiO_2_, were determined employing a QuAAtro Microflow Analyzer (SEAL Analytical, UK). The values obtained are graphically represented in Fig. 3 and tabulated in Table 2.

### DNA extraction and metagenome sequencing

DNA was extracted from membrane filters using a protocol based on manual cell lysis, followed by purification with the DNeasy Blood & Tissue Kit (Qiagen, Germany). The membranes were situated inside a 5 ml tube with the sample-filtered side inward. Subsequent to the addition of 1 ml of cell lysis buffer (20 mM EDTA, 50 mM Tris, 400 mM NaCl, and 0.75 M sucrose) and 5 μl of lysozyme solution (10 mg mL^−1^ in 10 mM Tris-Cl (pH 8.0)), tubes were incubated for 30 min at 37 °C in a horizontal orientation with a rotation speed of 5 rpm in a hybridization oven. Following this, proteinase K at a final concentration of 0.2 mg mL^−1^ and sodium dodecyl sulfate at a final concentration of 1% were introduced, and the tubes were further incubated at 55 °C overnight with rotation in a hybridization oven. After incubation, RNase A (200 μg mL^−1^), 1 mL of AL buffer (DNeasy Blood & Tissue Kit, Qiagen), and 70% ethanol were sequentially added to the tubes with appropriate incubation times. The manufacturer’s instructions of DNeasy Blood & Tissue Kit were adhered to from the stage of transferring the lysis mixture to the DNeasy Mini spin column. The quality and quantity of the extracted DNA were assessed using electrophoresis with 1% of agarose gel, Nanodrop ND-1000 (Thermo Fischer Scientific, USA), and Qubit 2.0 Fluorometer (Life Technologies, US) employing the Qubit® dsDNA Assay Kit. Metagenome sequencing was performed at Theragen Etex Inc. (Suwon, Korea). The Truseq library preparation kits with default library linkers and adaptors were used to generate sequencing libraries. The libraries were sequenced on an Illumina HiSeq 2500 platform, producing 250 bp paired-end reads.

### Taxonomic profiling of metagenomic reads

Raw sequence reads were decontaminated by adapter removal and quality trimming using BBDuk (v39.01) with the following parameters: ktrim = r, k = 23, mink = 11, hdist = 1, tpe, tbo, ftm = 5, qtrim = rl, trimq = 10, minlen = 100. Subsequently, the taxonomic profiling of these metagenomic reads was performed against a customized GTDB database (R207) generated by Struo2^34^ (http://ftp.tue.mpg.de/ebio/projects/struo2/GTDB_release207/). Taxonomic classification and species abundance estimation were performed using Kraken2 (v2.1.3) and Bracken (v2.7)^35^. The organization of the output report file was accomplished using Pavian^36^ (https://fbreitwieser.shinyapps.io/pavian/). Finally, the resulting species abundance information was visualized using the R package ‘tidyverse’.

## Data Records

This project has been deposited at DDBJ/ENA/GenBank under the SRP accession No. SRP396155^37^. The Sequence Read Archive (SRA) accession numbers associated with the metagenomes are available in Table 1.

## Technical Validation

**Fig. 4:**
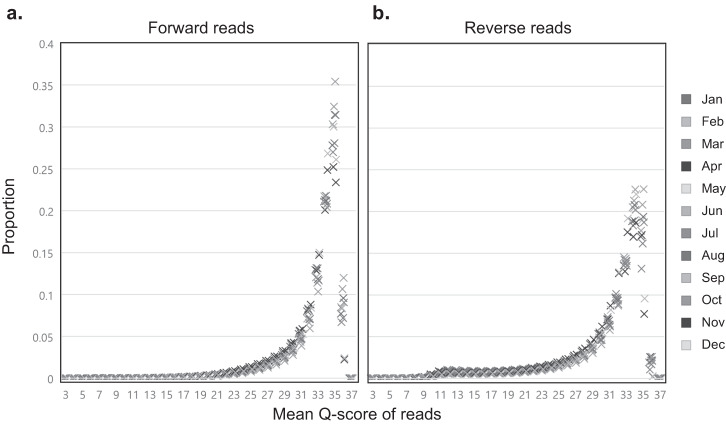
Distribution of per sequence mean quality scores of the 12 metagenomes.

**Fig. 5:**
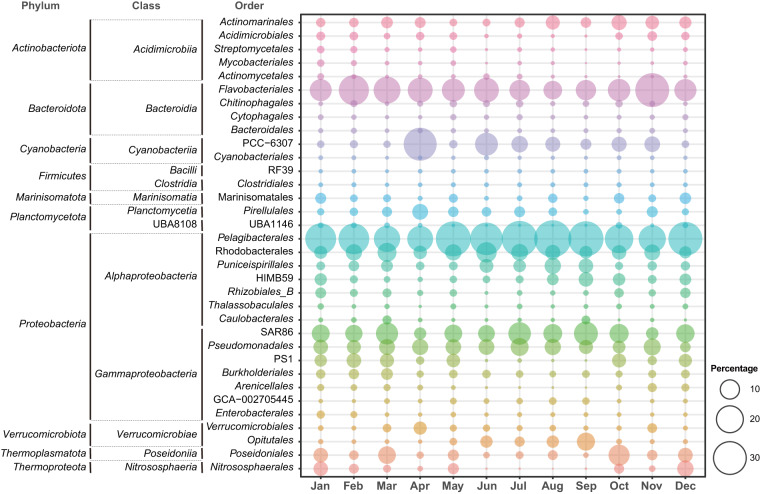
Taxonomic profiling of metagenomic reads retrieved from the East Sea. A total of 34 orders are presented that ranked in the top 30 by maximum or median among the 12 metagenome samples.

The assessment of quality scores for the raw reads of the 12 metagenomes was performed using FastQC (v0.10.1). The results show that ~91.88% and ~79.60% of the bases have quality scores of ≥20 and ≥30, respectively, indicating that sequencing was performed successfully (Fig. 4). The distribution of per-read quality scores across the 12 metagenomes was similar, further indicating no quality issues (Fig. 4). Consistent with the characteristics of the Illumina sequencing technology, the forward reads exhibited higher quality compared to the reverse reads (Fig. 4). A succinct taxonomic profiling analysis was then conducted to ascertain the suitability of the generated data for subsequent metagenomic analysis (Fig. 5). The taxonomic composition revealed a prominent dominance of *Pelagibacterales* (26.0%; a median value over a 12-month period), followed by *Flavobacteriales* (12.6%), SAR86 (7.7%), *Pseudomonadales* (5.5%), and *Rhodobacterales* (5.3%). These taxa are typically known for their prevalence in the ocean^1,4^. This observed pattern also aligns with the microbial community structure derived from culture-independent investigations conducted on a seawater sample collected from the same research station^31^.

## Data Availability

FastQC (v0.10.1) was used to check the quality of the raw data. No other code or software was used, as the original sequences were submitted.
